# A qualitative assessment of factors contributing to Spanish-speaking federally qualified health center patients’ chronic pain experiences

**DOI:** 10.1371/journal.pone.0285157

**Published:** 2023-05-18

**Authors:** Lauren Bifulco, Sarahí Almonte, Shantel Sosa, Leila Etemad, Destiny Ruiz, Mary L. Blankson

**Affiliations:** 1 Weitzman Institute, Community Health Center, Inc., Middletown, Connecticut, United States of America; 2 Department of Nursing, Community Health Center, Inc., Middletown, Connecticut, United States of America; 3 Department of Sociology, Wesleyan University, Middletown, Connecticut, United States of America; University of South Florida, UNITED STATES

## Abstract

People of Hispanic or Latino ethnicity (Latinx people) experience pain diagnosis, treatment, and care disparities relative to non-Latinx Whites. Those whose preferred language is Spanish may experience additional disparities when receiving care in a language-discordant environment. In order to better understand medically underserved Spanish-speaking Latinx patients’ pain care experience in primary care, we conducted semi-structured qualitative interviews with federally qualified health center staff members (n = 9) and Spanish-speaking adult Latinx patients with chronic pain (n = 12) to capture data on their perspectives. Interview data were mapped to the individual (microsystem), interpersonal (mesosystem), organizational (exosystem), and environmental (macrosystem) levels of Bronfenbrenner’s Ecological Systems Theory and analyzed using thematic content analysis informed by the Framework Method. Findings suggest that Spanish-speaking patients and English-speaking care team members may interpret information about pain state and severity differently, may have misaligned expectations about care, treatment methodologies, and treatment goals, and may experience difficulty forming a mutual understanding during health care encounters due to cross-linguistic and cross-cultural miscommunication. Patients preferred to describe their pain in words rather than with numbers or standardized scales, and both patients and frontline care team members expressed frustration with medical interpretation services, which added time and complexity to visits. Patients and health center staff emphasized the diversity of experiences among Spanish-speaking Latinx people, and the need to account for both linguistic and cultural differences during care encounters. Both groups supported hiring more Spanish-speaking, Latinx healthcare personnel who better resemble the patient population, which has the potential to improve linguistic and cultural concordance and competence, with the aim of improving care outcomes and patient satisfaction. Further study is warranted to examine how linguistic and cultural communication barriers impact pain assessment and treatment in primary care, the extent to which patients feel understood by their care teams, and their confidence in their ability to understand and interpret treatment recommendations.

## Introduction

Chronic pain is defined as pain lasting beyond the duration of normal healing time, and is often understood to persist for at least three to six months [1]. Approximately 20% of the adult U.S. population (~50 million people) meet criteria for chronic pain [2, 3], the majority of whom are treated in primary care [4, 5]. Increased risk for chronic pain is a public health challenge disproportionately impacting vulnerable and medically underserved patients, including those with low socioeconomic status, less than a high school education, and Medicare or Medicaid health insurance [6–9]. Despite federal policy initiatives dedicated to addressing disparities in pain care and treatment, racial and ethnic disparities persist [10]. These inequities include differences in how acute pain is assessed and treated in emergency departments and other outpatient settings [11, 12] for White patients versus patients of other races and ethnicities, racial differences in opioid prescribing and monitoring for chronic pain [13, 14], and unconscious bias in treating acute [11, 15–17] and chronic painful conditions across the lifespan [10, 16, 18–20].

The United States Census Bureau classifies individuals of any race who were born in or who have ancestry or nationality in Cuba, Mexico, Puerto Rico, South or Central America, or other Spanish cultures as Hispanic/Latino/Latina [21]. Here, we have adopted this broad definition, which embraces identity based on Spanish-speaking ancestry and/or geographic origin, and have operationalized the term “Latinx” to include people of Hispanic or Latino origin of any gender identity [22, 23]. Latinx people are more likely than non-Latinx Whites to experience chronic pain [2] but are less likely to receive pain assessment [11] and opioid analgesic treatment [11, 12, 24–26], which may indicate that their pain is not adequately understood or addressed [27].

Pain endurance and sensitivity can vary based on cultural expectations and norms for pain management [28–30]. When their pain is assessed during a care encounter, Latinx patients may experience difficulty expressing its state and severity. Some studies have found that Latinxs rate their pain severity higher than non-Latinx White patients in response to the same stimulus [31, 32], and that they endorse more severe pain and greater pain interference during clinical visits [30, 33]. However, Latinxs who identify with cultural values of stoicism or believe that ability to execute one’s daily tasks is an indicator of good health may only seek treatment once their pain reaches a high threshold [25, 34], and may be reluctant to accept medication as treatment [35]. Language’s social and cognitive influences [36] may also impact pain experience [37–39]. Spanish-speaking Latinx youth [28] and adults [40, 41] report less frequent musculoskeletal pain than English-speaking Latinxs when treated in English-dominant environments. A 2021 study of adult Latinx English-Spanish bilingual patients found that patients who identified their dominant linguistic and cultural identity as Hispanic/Latinx expressed more pain in response to an experimental stimulus when speaking Spanish versus English [29].

Though language-concordant care has been shown to increase patient satisfaction and improve pain care outcomes [42, 43], health system-level and workforce barriers to adequate pain-related communication between care teams and patients whose primary language is Spanish (Spanish-preferred patients) include time and resource constraints that limit mutual understanding [23, 41]. Health care teams report that insufficient language services, including limited availability of live interpretation and translated materials, often present barriers to equal care and treatment [44, 45], and Spanish-preferred patients confirm missed opportunities for clear patient-provider communication, and inability to fully understand their treatment options [35].

Federally Qualified Health Centers (FQHCs) [46] are non-profit organizations that care for approximately 28 million vulnerable and medically underserved patients in the U.S. health care safety-net [47]. More than a third are served in a language other than English [48]. Patients receiving care in FQHCs are often uninsured or publicly insured, and are likely to be members of vulnerable populations, including racial and ethnic minority groups, and/or medically underserved populations such as those with below average socioeconomic status, educational attainment, and/or access to healthcare services [46]. In September 2018, Community Health Center, Inc., a statewide, multi-site FQHC in Connecticut, implemented a new two-step process to screen for chronic pain and administer a chronic pain functional assessment measure to those who screened positive [49]. Approximately 4200 Latinx patients screened positive for chronic pain during the year following initial implementation, of whom approximately 1800 indicated Spanish as their preferred language. Over two-thirds of Latinx patients with positive chronic pain screening and two-thirds of patients with Spanish as their preferred language reported severe pain and severe pain interference with function (≥7 on a 0–10 scale). To better understand these findings, we conducted a qualitative study, guided by Bronfenbrenner’s Ecological Systems Theory framework [50] to explore potential contributory factors related to patients’ experience and their interactions with health care providers and care teams, health care systems, and the health policy landscape. Based on published literature, we hypothesized that both Spanish-speaking patients with chronic pain and health center staff would perceive the potential for improvement in pain care delivery across each of these domains, but would feel unprepared to address difficulties in cross-linguistic and cross-cultural communication.

## Materials and methods

### Study setting

Community Health Center, Inc. (CHCI) is a large, statewide FQHC in Connecticut that serves as the primary care medical home for 145,000 patients. Two-thirds of CHCI patients are Medicaid-insured, the majority are racial and ethnic minorities, and 90% of patients have income at or below 200% federal poverty level. Approximately 48% of CHCI’s adult primary care patients are Latinx, and 26% have Spanish documented as their preferred language.

### Participants

We conducted semi-structured interviews with 9 FQHC staff (clinical care team members, administrative leaders and non-clinical staff), and 12 Spanish-speaking adult primary care medical patients with chronic pain. FQHC staff were recruited to represent diversity in location, job role, demographics, and Spanish proficiency, and patients were purposively sampled to reflect a variety of care delivery locations, pain complaints, and demographic characteristics. Patients were eligible for inclusion if they were age 18+, had Spanish listed as their preferred language in the electronic health record, had screened positive for chronic pain during a primary care visit conducted between 3/1/20 and 9/30/20 by reporting pain “most days” or “every day” within the past 3 months [2, 49, 51], and had been administered the 3-question PEG pain interference measure [52] (pain score, interference with enjoyment of life, and interference with general activity), which is assessed on a 0–10 scale, with scores of 7 or higher indicating severe interference with activities of daily living.

### Measures

#### Theoretical framework

We used a constructivist analysis paradigm [53, 54], to develop semi-structured interview questions that reflected the four interacting, hierarchical, ecological levels of Bronfenbrenner’s (1979) Ecological Systems Theory–the microsystem (the individual), the mesosystem (interpersonal interactions and relationships), the exosystem (the individual’s surroundings and context and circumstances that impact interpersonal interactions), and the macrosystem (broad contexts and circumstances present in society such as laws, conventions, beliefs, and cultural norms) [50]. We adapted this framework for use in health services research by mapping its levels to the patient (microsystem), care team (mesosystem), organization (exosystem) and environment (macrosystem) when developing the interview guides for FQHC staff and patients.

#### Interviews

We conducted thirty-minute videoconference interviews with FQHC staff between November 2020 and January 2021, in English, using a semi-structured interview guide, which included a demographic questionnaire (S1 Appendix). Teams of two researchers, led by a bilingual Latinx nurse-researcher, conducted fifteen-minute telephone interviews with patients with chronic pain between January and March 2021, in Spanish, using a separate semi-structured interview guide (S1 Appendix). All interviews were recorded and transcribed verbatim for analysis, and Spanish-language patient interviews were translated to English by the bilingual, bicultural researcher who had led them. To minimize ambiguity, translation from Spanish to English was done based on meaning rather than literal interpretation [55, 56] and the research team had access to both the verbatim Spanish transcripts and the English translations.

We followed the Standards for Reporting Qualitative Research (SRQR) reporting guidelines for qualitative studies [57]. The Community Health Center, Inc. Institutional Review Board approved the study protocols for staff interviews (Protocol #1181, 11/12/2020) and patient interviews (Protocol #1182, 11/23/2020). FQHC staff and patients provided verbal informed consent to participate.

### Analyses

Informed by the Framework Method of thematic analysis [58, 59], we developed working definitions for each ecological level (microsystem/”individual”, mesosystem/”interpersonal”, exosystem/”organizational”, macrosystem/”systems and policy”) and identified themes and subthemes under each domain through inductive content analysis of interview transcripts [60]. A team of four researchers independently coded each transcript, and assigned segments of text to the appropriate theme and subtheme. All researchers who co-developed the coding schema and who coded transcripts were female and proficient or fluent in English and Spanish; two were native/heritage Spanish-speakers who self-identified as Latinx. Coding discrepancies were discussed and resolved, with the bilingual Latinx researcher/licensed clinical staff member who had led the patient interviews providing insight in order to break ties. NVivo version 12 (Sydney, Australia), and Microsoft Excel were used to manage and analyze data.

## Results

### Participant characteristics

#### FQHC staff interviews

FQHC staff participants (n = 9) routinely worked at 10 care delivery sites, and had worked at the health center for an average of 5.4 years. They averaged 36.5 years old, were mostly female-identifying (n = 8) and the majority (n = 8) had an advanced degree, with 6 participants holding terminal academic or medical degrees (e.g. MD, PhD). About half of the participants were non-White (n = 5), and two reported more than one race or ethnicity. Four participants self-identified as Latinx, most of whom (75%) reported Puerto Rican ethnicity. On average, participants self-rated their Spanish proficiency as 3.6/5.0 on a 0–5 Likert scale, indicating that they spoke and understood enough Spanish to express themselves in professional settings. Three participants spoke Spanish as their first language, and two participants reported understanding only basic Spanish. Table 1 shows the FQHC roles held by the staff members interviewed.

**Table 1:** Staff interview participants (n = 9).

Role	Attributes
Senior Administrative Leader (Clinical) (n = 2)	• Provides direct patient care (n = 2)
	• Organizational expertise (n = 2)
	• Decisional Authority for Organization (n = 2)
	• Health Policy expertise (n = 2)
	• Fluent in English, Basic Spanish only (n = 2)
Senior Administrative Leader (Non-Clinical) (n = 1)	• Organizational expertise
	• Decisional Authority for Department
	• Health Policy expertise
	• Fluent in English and Spanish
Site-Level Leader (Clinical) (n = 1)	• Provides direct patient care
	• Organizational expertise
	• Operational expertise at clinical site
	• Decisional authority at clinical site
	*•* Health Policy expertise
	• Fluent in English and Spanish
Primary Care Provider (n = 2)	• Provides direct patient care (n = 2)
	• >50% visits in Spanish (n = 2)
	• Fluent in English and Spanish (n = 2)
Behavioral Health Provider (n = 2)	• Provides direct patient care (n = 2)
	• >50% visits in Spanish (n = 1)
	• Fluent in English and Spanish (n = 1)
	• Fluent in English/Intermediate Spanish (n = 1)
	• Lived experience receiving primary care in English and Spanish (n = 1)
Non-Clinical Health Center Team Member (n = 1)	• Health Policy expertise
	• Fluent in English and Spanish
	• Lived experience receiving primary care in English and Spanish

#### Interviews with patients with chronic pain

We placed phone calls to 32 patients aged 18+ with Spanish listed as their preferred language in the electronic health record who had screened positive for chronic pain and completed the PEG pain interference measure between 3/1/2020 and 9/1/2020. Fifteen patients (46.9%) consented to be interviewed and confirmed that they experienced chronic pain. We excluded three interviews from our analysis where the participant endorsed acute pain at the time of the call as their only pain complaint (n = 1), and where the participant endorsed chronic pain but was unwilling or unable to discuss their history of chronic pain with the interviewers (n = 2).

Interviewed patients (n = 12) received care at eight different primary care sites, and half (n = 6) were Medicaid-insured. All had screened positive for chronic pain at their most recent visit (self-reported pain “Most Days” or “Every Day”), with nearly all reporting pain “Every Day” (n = 11). The mean score on the PEG pain interference measure was 6.9/10.0 (n = 12), though the majority of patients (n = 9) recorded a severe average score (≥7.0/10.0). All participants self-identified as Latinx (n = 12), a slight majority were male (n = 7), and the average age was 52.5 years. Demographic information for interviewed patients is summarized in Table 2.

**Table 2:** Demographic characteristics of patients interviewed (n = 12).

Characteristic	N(%) or *M*(*SD*)
	(n = 12)
**Age**	
30–39	2 (16.7%)
40–49	3 (25.0%)
50–59	4 (33.3%)
60–69	1 (8.3%)
≥70	2 (16.7%)
**Gender**	
Male	7 (58.3%)
Female	5 (41.7%)
**Race**	
Hispanic/Latinx	11 (91.7%)
White	1 (8.3%)
**Ethnicity**	
Hispanic/Latinx	12 (100%)
**Insurance**	
Medicaid	6 (50.0%)
Medicare	3 (25.0%)
Private	2 (16.7%)
Uninsured	1 (8.3%)
**PEG scores (n = 12)**	
Pain intensity (P)	*6*.*8(2*.*7)*
Severe score (≥7.0)	9 (75.0%)
Interference with enjoyment of life (E)	*7*.*0(3*.*1)*
Severe score (≥7.0)	9 (75.0%)
Interference with general activity (G)	*6*.*8(3*.*1)*
Severe score (≥7.0)	8 (66.7%)
Total PEG	*6*.*9(2*.*9)*
Severe score (≥7.0)	9 (75.0%)

Table 3 shows themes that emerged during interviews with FQHC staff and patients, organized by ecological framework domain. S2 Appendix shows additional quotes illustrating each theme.

**Table 3:** Interview themes.

Ecological Framework Domain	Operationalized As	Themes and Subthemes
**Microsystem: Patient Level**	The interviewee’s own beliefs, values, attitudes, knowledge, and behaviors	***Patients’ Pain Experience***
		Attitude towards Treatment
		Home Remedies
		Pain and Behavioral Health
		***Expressing Pain***
		Acknowledging Physical Pain
		Difficulty Locating or Describing Pain
		Vocabulary of Pain
**Mesosystem: Care Team Level**	The interviewee’s perception of interactions between patient and staff member regarding pain care, and patients’ beliefs, values, attitudes, knowledge	***Empathy***
		***Mutual Understanding***
		Alignment between Patient and Provider
		Cross-Linguistic Communication
**Exosystem: Organization-Level**	The interviewee’s experience of how patients with chronic pain are cared for at the organization	***Improving Access and Quality through Resources and Staffing***
		Interpretation Services
		Spanish-Speaking Personnel
		Time Constraints
**Macrosystem: Systems and Policy Level**	The interviewee’s knowledge of and perspectives on the influence of health policy and social determinants of health on how patients are cared for	***Approach to Pain Treatment***
		***Culture***
		Diversity among Spanish-Speaking Patients
		Need for Linguistic and Cultural Concordance and Competence
		***Social Determinants of Health***
		Financial, Occupational, and Social Stressors
		Health Disparities
		Information and Resources

### Microsystem/patient-level

#### Patients’ pain experience

Patients expressed frustration that their chronic pain had not been completely alleviated by treatment. Many expressed sadness, futility, or anger, and several endorsed the use of home remedies concurrent with prescribed treatment, or reliance on “God” to help manage or alleviate pain. A patient stated *“Estoy sufriendo con la rodilla*, *mi cadera*, *y mi espalda*. *Me estoy poniendo vieja*. *No hay nada que puedo hacer*. *Entonces yo qué hago*, *algunas veces no aguanto el dolor*, *me quedo en la cama y lloro*. *Es lo que puedo hacer*.*”* (“I’m suffering with my knee, my hip, my back. I’m getting old. There is nothing else I can do. So what do I do, sometimes I can’t take the pain, I stay in my bed and just cry. It is what I can do”) [Patient 12; P12].

Patients who reported using home remedies in addition to what their doctor had recommended also reported limited success alleviating their pain. A patient who had a history of multi-site joint pain and orthopedic surgery stated: *“Ya tú sabes que yo soy Puertorriqueña y cojo el [name-brand topical pain relief product] pero sufro del dolor*. *Me pongo el [topical pain relief product]*, *me tomo mucho té y a lot of ‘rubbing ointments’*, *[rubbing] alcohol*, *[name-brand mentholated topical ointment] y hielo*, *pero no puedo hacer más nada*.*”* (“Well, you already know that I am Puerto Rican and I use [name-brand topical pain relief product] but still suffer from pain. I rub the [topical pain relief product] and I drink a lot of tea and use a lot of rubbing ointments, [rubbing] alcohol, [name-brand mentholated topical ointment], and ice, but I cannot do anything more”) [P12].

#### Expressing pain

Clinical staff’s description of their Spanish-speaking patients with chronic pain aligned with patients’ description of their own experiences. Several clinicians noted that their Spanish-speaking patients with chronic pain regularly report multiple pain complaints, diffuse or widespread pain, and high pain scores, and it can be difficult to make appropriate care recommendations. A bilingual clinician stated, “[Patients say] ’I have a *molestia*’… And it’s hard sometimes for them to put into words. It’s [translated as] ‘something’s bothering me’ or ‘it’s this annoying bothersome thing’ but no [descriptors like] ’dull ache’, that sort of thing" [S6]. A second bilingual clinician commented, “[Spanish-speaking patients] are more descriptive in terms of using metaphors, like *‘El dolor que me quiere arrancar la vida’* (‘Pain that wants to rip the life out of me’) or they describe [pain] using a comparison with an animal, for example: *‘Hoy tengo el mono atrapado’* (’Today I have a trapped monkey’”) [S9].

Patients’ use of non-quantifiable terms, metaphors, and comparisons was challenging for primary care teams, who often use patient-provided descriptors and scores to better understand overall health status and guide treatment recommendations. Though pain assessment and care at the FQHC emphasizes the biopsychosocial nature of pain, most patients seemed to view their physical pain as separate from other biopsychosocial factors, and few acknowledged behavioral health comorbidities that contributed to or co-occurred with their pain. One patient felt that her provider had incorrectly assumed she needed treatment for depression and anxiety in conjunction with pain: *“Yo nunca padecí de depresión ni ansiedad ni nada de eso y ahora estoy tomando como cinco pastillas para ansiedad y depresión… Los doctores te dicen ‘tienes esto’ pero cómo es que ellos no se meten en el cuerpo de uno y pensar que saben que le ha afectado a uno*?*”* (“I never suffered from depression or anxiety or anything like that and now I’m taking about five pills for anxiety and depression. The doctors tell you ’you have this’ but how is it that they don’t get into one’s body and they think that they know what has affected you?”) [P2]. Later in the interview, when asked what aspects of her treatment were helpful, the patient acknowledged that behavioral health treatment had helped her pain: *“Yo venía de muchos doctores*. *Pero en realidad… la que ha venido un poquito a ayudarme es la terapista y la psicóloga*, *ok*?*”* (“I went to many doctors. But in reality what helped me a little is the therapist and the psychologist, ok?”) [P2].

Care team members espoused the importance of assessing how a patient’s mental health impacted their pain, but several expressed concern that Spanish-preferred patients might omit information or avoid discussing their mental health status and any contributing events. A clinician commented that in her experience, not all patients feel secure enough to disclose information about psychosocial distress with their care team: "Depending on where someone is from in the Spanish-speaking world, in a scenario where maybe it wasn’t safe to talk about depression or anxiety or something like that, sometimes what will come out is more of a physical complaint. So I think teasing that out sometimes can be difficult" [S5].

### Mesosystem/care team-level: Patient-provider communication about pain

#### Empathy

Patients recommended that frontline care teams express greater empathy towards their patients with pain. A patient stated: *“Como te digo*, *yo entiendo que los doctores te dan cualquier pastilla p’al dolor pero en realidad*, *pues*, *ellos no le importan lo que uno está sufriendo…*. *No le dan importancia a los síntomas de la gente* …*”* (“Like I tell you, I understand that the doctors will give you any type of pill for pain, but in reality they don’t care if one is suffering… They don’t care about symptoms people have…”) [P6]. A second patient corroborated this frustration when discussing how clinicians did not seem receptive to the information he provided when asked about his pain: *“Mira*, *para ser franco es que no quieren entenderme verdad porque yo sé que yo tengo y a veces ellos malentienden*. *Ellos no saben lo que yo siento*. *Yo le expreso a mi doctor*, *pero no sé*. *No me [han] ayudado*.*”* (“Look, to be frank it is that they don’t want to understand me, honestly, because I know what I have and at times they misunderstand me. They don’t know what I feel. I express [this] to my doctor, but I don’t know. [They] have not helped me“) [P9].

#### Mutual understanding

Care team members’ perceived lack of empathy may be partially explained by misalignment between patient and provider regarding how to discuss pain during a clinical encounter. Communication between patients and their care teams about pain often starts with the patient being asked to rate their pain level. A bilingual clinician commented that in her experience, Spanish-speaking patients prefer not to use a 0–10 pain scale to describe their pain, stating, “Many have said that they feel *‘tonto’* [‘silly’] saying a number because they feel that a number doesn’t sufficiently describe the pain they feel” [S9].

A non-clinical staff member who is a native Spanish speaker posited that giving a high pain score helps patients signal the importance of their complaint to their care team, stating: “I am bothered or frustrated when [my] doctor says ‘on a scale of 0 to 10…’ How? Just please treat me! … If I say 8, they’re gonna prioritize something else… do I always have to say 10? … In Spanish I can say ‘it’s a 6, but this is what’s happening’. But in English, I might say it’s a 6 and then not really be able to describe what it really is” [S3].

Despite some misalignment between how patients and their care teams characterized, described, and understood pain, most patients interviewed were pleased with the relationships they had developed with their primary care provider and care team. A patient attributed her positive relationship with her (Spanish-speaking) primary care provider to feeling understood: *“Me siento cómodo porque mi proveedor… es muy comprehensiva y me entiende*. *Me da tiempo*, *toma tiempo de escuchar lo que me pasa*.*”* (“I feel comfortable because my doctor… is very understanding and understands me. She gives me time, takes time to understand what is happening to me”) [P1].

### Exosystem/organization-level

#### Improving access and quality through resources and staffing

Staff members and patients expressed that the language translation resources available to them made communication possible, but that translation services are not sufficient to foster personal connections with patients. A clinical staff member observed: "You can’t get any less personal than [the telephone translation service used at the FQHC]. There’s no human connection there, literally… In order for you to have any true impact on a patient’s life, in my experience, you have to build that relationship" [S2]. A second clinical team member reflected that when given a choice, some patients decline translation services, stating, “Most of my patients would rather [I use] my broken Spanish than use a translator. My conversations… [are] more nuanced and can be more about forming a connection with my patient that’s kind of casual—casual conversations with the translator can be tough" [S7].

A patient expressed that she hesitated to ask for a translator unless absolutely necessary due to uncertainty about making an “extra” request of her care team: *“Cuando yo siento que necesito hablarle [al proveedor] algo que me este preocupando estoy preocupado que [estoy pidiendo algo]”extra” si pido traductor para poder explicarlo con el doctor*.*”* (“When I feel like I have to talk to [the provider] about something that’s bothering me, I’m worrying that [I’m asking for something] “extra” if I ask for a translator so that I can explain myself to the doctor”) [P2].

FQHC staff also felt conflicted about how using translation resources impacted their visits with Spanish-preferred patients, but were concerned about whether they were able to do enough to make patients feel comfortable and meet their needs. A clinician remarked on the challenge of having the same amount of time with each patient regardless of whether translation is needed: “My clinic could run like an hour behind… I don’t want to short-change [patients] just because I’m using an interpreter—I want to connect in the same way that I connect with my English-speaking patients” [S1]. A second clinician remarked that some patients were already frustrated at not having more visit time with their clinical team, and felt that using a translation service made the visit time seem even shorter: “[Patients will say] ’I don’t even know why I come here! I only see you for 5 minutes and then you charge my insurance!’ [Patients will] tell the provider just like that” [S2].

When asked what the FQHC might do to better meet patient needs, patients expressed that they would appreciate more bilingual care team members: *“Sería buena que las clínicas tuvieran más personas bilingües porque unas personas tienen problema hablando en ingles y nada mas hablan español”* (It would be great if the clinics had more bilingual people because some people have trouble speaking in English and only speak Spanish”) [P1]. FQHC staff recommended modifying hiring practices with respect to both language and culture. An administrative leader stated: “There’s something to be said of recruiting people who are representative of the community that we serve… [and] intentional recruitment in the communities or neighborhoods where [FQHC] is located” [S8]. Staff recommended salary incentives as potential motivation for care team members to build their Spanish competence. An administrative leader posited, “Maybe [FQHC] should open some specific Spanish-speaking positions where [we] will only take a bilingual person and then actually pay them additional because of that, because it’s such a valuable asset…” [S5].

### Macrosystem/systems and policy level

#### Approach to pain treatment

Clinicians noted that some Spanish-preferred patients who had previously received care for their pain outside of the mainland U.S. had developed treatment expectations that differ from current standards in place at the FQHC, often leading to difficult conversations. A behavioral health provider remarked on such patients’ expectations about seeking care for pain: “If we are defining Spanish-speaking patients as a whole culture, like being Hispanic or Latino, the tolerance of pain could be lower than other cultures, [with] more focus on relieving physical pain [and] discomfort right away… They are thinking that I’m going to prescribe a medication to relieve their pain so I have to clarify… explaining again the connection between chronic pain and mental health" [S9]. A primary care provider observed that patients may also expect medications, dosages, and treatment plans that are not aligned with the FQHC’s prescribing policies or with guideline-informed pain care: "Prescribing practices around opioids are really different [outside the mainland U.S.]… patients come over on, like, long-term [opioid analgesics]… Per current guidelines, that’s not really indicated for chronic pain that’s non-cancer-related… It’s really hard to navigate that conversation, because what do you do when someone’s been on this medication for 10 years?" [S5].

#### Culture

When discussing recommendations for health center policy changes to improve pain care for Spanish-speaking patients, staff recognized that the FQHC’s Latinx patients come from diverse cultural backgrounds, and that it is not appropriate to assume that linguistic competence corresponds to cultural understanding. A non-clinical staff member and native Spanish speaker advocated for placing equal emphasis on linguistic proficiency and cultural competence, reflecting that, “You still want to provide bilingual bicultural services… we need to understand that the culture is different, and it’s not that one is less than the other… We need to make sure that we appreciate as much as we can, empathize with them, and then at the same time make sure that we are also able to contribute rather than impose" [S3]. A bilingual clinician who had worked in primary care outside the mainland U.S. suggested offering more treatment alternatives designed to appeal to patients’ cultural experiences and preferences: “A lot of people who are native Spanish speakers come from more collectivist cultures. And often, group care, like group visits, can work well …And I think in some settings, yes, that it’d be cool if we had more Spanish-language chronic pain support groups for patients" [S4].

#### Social determinants of health

Staff members discussed the connection between pain, stress, trauma, and the contributory factors beyond the control of the healthcare system that might impact pain care experience and ability to follow care recommendations. A clinician observed: “[FQHC’s racial, ethnic, and linguistic] minority patients certainly have a higher percentage of economic hardships, social hardships, and just other social stressors in their life—food insecurity, transportation insecurity … Even decreased access to basic things like medical care” [S4]. A non-clinical staff member identified unequal information access as an additional source of disparity, stating, “The Spanish-speaking community might be segregated or secluded–not only geographically, but also in terms of knowledge about community resources” [S3]. A second staff member commented on the downstream impact of information and access disparities on pain severity: “By the time they come to a healthcare professional’s attention [the pain] may already [be] severe, right? Like a pain that they’ve been struggling with for years. So it goes back to those social root causes” [S8].

Both patients and staff recognized lack of financial resources and loss of income as major barriers to adhering to pain care recommendations. A primary care provider recalled a patient with severe pain who worked physically demanding day labor jobs and repeatedly declined additional testing, since he feared that both the testing and the result might cause him to miss work and lose income: "He kept telling me, ’I’m fine’, because he wanted to keep working. And I insisted he get an x-ray, but it was a big to-do to convince him, because of financial and occupational stressors” [S5]. When asked how it felt to speak with a primary care provider and care team about pain, a patient shifted the conversation to discuss how their care team had been unable to help her keep working despite her pain: *“Básicamente ellos no te ponen atención… yo tuve que pedirle favores al doctor que me diera de baja en el trabajo porque yo tenía de 5 días de trabajo me lo pasé para 4 [días] a 32 horas y de 32 horas baje a 3 días*. *Y de los 3 días de trabajo que tenía casi siempre estaba ‘calling out’… Llevo 25 años viviendo en los Estados Unidos y mi plan era*, *pues como todo hispano*, *venir aquí a trabajar y salir adelante*.*”* (Basically, they don’t pay attention to you… I had to ask for favors from the doctor, to decrease work because I had 5 days of work, I reduced to 4 [days] to 32 hours, and from 32 hours I went down to 3 days. And of the 3 days of work that I had, I was almost always ’calling out’… I have spent 25 years living in the United States and my plan was, like all Hispanics, I came here to work and leave ahead”) [P2].

## Discussion

Pain is among the most common reasons for seeking primary care treatment [61], is subjective, and is influenced by the patient’s lived experience. Managing and treating pain is difficult, and there are few universal guidelines available to serve as a roadmap [62]. The United States National Pain Strategy calls for improved communication between patients and providers about pain, and pain treatment that accounts for patient preferences alongside evidence on safety and effectiveness [4]. Language discordance between patient and care team further magnifies the challenge of establishing the patient-provider relationship, achieving mutual understanding, and incorporating patient preferences into care.

In our sample, clinical staff members noted nuances in idioms, terminology, and ways that Spanish-speaking patients commonly discussed their pain. Staff members acknowledged the need to navigate cultural and linguistic barriers, and the persistent challenge of relieving chronic pain. Patients expressed both desire for more pain relief and passivity and fatalism with regard to their pain. They commented on the difficulty of being understood during care encounters, and the importance of connection with their care teams. Several who were satisfied cited feeling understood by their provider and care team as a reason they were happy with their care.

The United States Department of Health and Human Services’ Health Communication objectives for Healthy People 2030 include increasing the number of adults with limited English proficiency (LEP) whose healthcare providers offer explanations that are easy for patients to understand [63]. The U.S. Census Bureau’s American Community Survey found that in 2019, over one fifth (22.0%) of United States residents spoke a language other than English at home [64]. Though approximately two-thirds of Latinx people in the United States are proficient English speakers or are bilingual in English and Spanish, a significant number of Latinx people are monolingual Spanish-speakers and/or report LEP [65]. Language proficiency has emerged as a factor that interacts with other determinants of health and healthcare access for Latinx patients, and care disparities persist despite legal and health policy protections, informational resources on improving communication with LEP patients [66], and widespread understanding of the value of trained interpreters in improving quality of care. Patients report stigma and anxiety associated with language-discordant healthcare encounters [67], and note that they sometimes feel pressured to settle for care teams with limited language skills, feel embarrassment, and fear discrimination associated with the perception that their healthcare encounters create extra work, or with voicing concerns about communication difficulties [42].

Differing approaches to pain treatment add to the friction between patients and clinicians during pain-related encounters. From clinicians’ perspectives, the general goal of pain treatment is more likely to be measurable improvement than complete pain relief [68]. Numeric or categorical pain assessment scales are frequently used to establish a basis for measurement and comparison over time. Both patients and clinicians in our sample perceived a disconnect between how patients described their pain in words and clinicians’ need to operationalize and translate these words to gauge pain on 0–10 scale and assess its nature and location. These mismatched expectations led to a chasm in mutual understanding of patients’ pain. Mutual understanding and acceptance of any pain scale is difficult given that pain is subjective and pain experience is highly personal. Decision support tools have been proposed that will use patient-provided information to facilitate communication with providers around pain experience, goals, preferences, and functional status [69]. Tools that prompt shared decision-making about treatment plans and goals may help care teams build patients’ buy-in and comfort by making sure their concerns are heard, and may help clarify expectations around pain relief [70].

Staff interview participants recognized that sociodemographic factors and unequal information and access might impact Spanish-preferred patients’ pain care experience and ability to follow care recommendations. Latinx people in the U.S. are less likely than non-Latinx people to utilize and access health services or to have a usual source of care, and are over twice as likely to be uninsured as non-Latinx Whites [71]. Compared to non-Latinx Whites, Latinx people experience greater socioeconomic disparities in income, poverty rates, educational attainment, and geographic access to health services [72]. U.S. Latinx patients who speak Spanish as their primary language face additional barriers, as English proficiency has been identified as a strong predictor of health literacy, the ability to find, understand, and use health information that informs health decisions [73–75], and low health literacy is associated with worse health outcomes [73, 76]. Collecting and leveraging data on social determinants of health–the individual, environmental, and social factors that impact health and the ability to receive healthcare–can help identify and mitigate care disparities based on factors such as race, ethnicity, and language, and help shape FQHC policy around providing culturally competent treatment.

FQHCs should also strive to recruit, hire, and retain personnel who share culture and language with the diverse patient populations they serve. Patient-physician racial concordance is associated with higher levels of patient satisfaction about clinical communication [77]. Some published evidence indicates that patients’ perceptions of clinician empathy can positively impact their self-rated pain intensity and health-related quality of life [78]. In our study, both patients and FQHC staff highlighted the value of hiring clinical team members who reflect the patient populations they serve, and speculated that this could lead to deeper understanding of and empathy with patients’ needs. A study by Bloch (2017) proposes that Latinx nurses may be more perceptive of Latinx patients’ pain, suggesting that Latinx people may be better able to make pain assessment and management decisions for their Latinx patients [79]. Ng et al (2019) found that Latinx-American young adult volunteers presented with pain care vignettes were more sensitive to patients’ pain levels than their White American counterparts who were presented with the same vignettes, and ascribed a higher average pain level to each case [25]. However, Latinx patients in most areas of the United States are unlikely to be cared for by a Latinx team; approximately 6% of physicians [80] and 8% of nurses [81] identify as Latinx.

Our investigation was intended to help improve our organization’s understanding of patients’ and care team members’ experience during language-discordant pain care encounters. Further study is warranted to examine how linguistic and cultural communication barriers impact pain assessment and treatment in primary care, the extent to which patients feel understood by their care teams, and their confidence in their ability to understand and interpret treatment recommendations. Findings from this study will be used by clinical and administrative leaders to help update policies and procedures for asking patients about pain and function during primary care visits. As an organization that strives to deliver healthcare in the most equitable way, we recognize that the patient perspective is valuable in creating and co-creating knowledge that improves clinical care. More broadly, these findings are intended to shed light on patients’ lived experience and to draw frontline care providers’ attention to the many aspects of patients’ social identities–including language and culture–that can impact their pain care experience.

### Limitations

We did not explicitly ask patients about their immigration status, which may have limited our understanding of the context of their responses. Our study was set in an FQHC that cares for patients regardless of their documentation status, and does not collect immigration status or documentation status information at intake, which may have helped mitigate patients’ concerns about disclosing their status if it was relevant to their experience. Due to small sample size and unavailability of data on patients’ country of origin, we were not able to compare patients’ responses based on country or region of origin.

Due to restrictions imposed by the COVID-19 pandemic, we were unable to conduct in-person interviews with patients and staff, which may have impacted rapport between interviewers and interviewees. Further research examining the needs of Spanish-speaking patients with pain should utilize in-person interview methods to mitigate potential communication barriers imposed by use of telephone or videoconference, and should consider expanding the interviews [82]. Though peer-reviewed literature suggests that Latinx patients may ascribe religious or spiritual meaning to pain and suffering, may believe that they are being punished or tested when experiencing pain, and may pursue religious coping strategies such as prayer or counseling from a religious leader [27, 83], it was beyond the scope of the study to analyze patients’ perceptions of the factors beyond their diagnosis and physical symptoms that contributed to their pain onset, persistence, severity, and somatization. Additional research is warranted to examine the impact of patients’ and cultural groups’ beliefs about pain on their perception of their pain and pain care.

The scope of our study did not permit direct comparison between monolingual or LEP Spanish-speaking patients with chronic pain and patients with stronger comprehension of English, who may feel more comfortable using at least some English during a care encounter. Future work will aim to better understand differences between how monolingual and LEP Spanish-speaking patients and patients who are comfortable using at least some English during medical visits interpret questions about “pain” from their care teams, interpret questions about how pain impacts their lives, communicate their needs to their provider and care team, feel that those needs are understood and met, and understand and interpret treatment recommendations.

### Conclusions

Current literature has demonstrated disparities between English-speaking and Spanish-speaking Latinx patients in reporting pain, seeking pain care, and receiving treatment. This study adds to the body of knowledge on how to develop and refine patient-centered pain care practices to meet the needs of medically vulnerable Spanish-language-preferred primary care patients. We advocate for including the voices of patients and care teams to guide formulation of chronic pain screening and follow-up policies.

## Supporting information

S1 Appendix Interview guides. (DOCX)

S2 Appendix Themes and example quotations from FQHC staff and patient interviews. (DOCX)
