# Supplementary material for: A qualitative assessment of factors contributing to Spanish-speaking federally qualified health center patients’ chronic pain experiences
Source: PLoS One. 2023 May 18;18(5):e0285157. doi: 10.1371/journal.pone.0285157 (PMC10194871; doi:10.1371/journal.pone.0285157)
Supplement: S1 Appendix — (DOCX) [file pone.0285157.s001.docx]

## **Appendix 1: Interview guides**

## **Clinical and administrative leader and care team member interview guide**

Interviews assessed staff’s personal perspectives regarding what Spanish-preferred patients experience when seeking pain care (microsystem), their interpersonal interactions with Spanish-preferred patients with pain (mesosystem), and policies and practices in place at the health center (exosystem) and in the external environment (macrosystem) that impact these patients’ care experience.

DEMOGRAPHICS

1. What is your role at the health center?
2. How long have you worked at the health center? (years, months)
3. Which health center site(s) do you work at?
4. How old are you:
5. What degree(s) do you hold:
6. Do you think of yourself as:

Male
Female
Transgender Male, Trans Man, Female to Male (FTM)
Transgender Female, Trans Woman, Male to Female (MTF)
Genderqueer (neither exclusively male nor female)
Other gender category, please specify__________________
Don’t know
Choose not to disclose

1. What race and ethnicity do you identify as: (Select all that apply)

White

Black or African American

Asian

Native American

Native Alaskan

Pacific Islander or Native Hawaiian

Other ________________

1. Do you identify as Hispanic or Latinx? Yes No
   If so, do you identity as Mexican, Puerto Rican, Cuban, Dominican, Central or South American, Caribbean or some other Latin American background? (Select all that apply)^[[1]](#footnote-1)^
2. On a scale of 1-5, how proficient are you at speaking Spanish?^[[2]](#footnote-2)^

I can read, speak, and understand…

- 1 – Only basic Spanish words
- 2 – Enough Spanish to have simple conversations at home or work
- 3 – Enough Spanish to participate in most conversations on practical, social, and
   professional topics
- 4 – Enough Spanish to express myself proficiently in any professional setting
- 5a – I am a native speaker
- 5b - I read, speak, and understand Spanish as well as an educated native speaker

**Open-Ended Questions:**

1. What has the health center done to meet the needs of Spanish-language-preferred patients?
2. What do you feel that your site or the organization can do to improve care for Spanish-language-preferred patients?
3. Why might chronic pain be especially prevalent among medically underserved patients in the U.S. who are racial and ethnic minorities?
4. How might a patient’s lived experience and culture impact how they experience or express pain?
5. Are there any structural barriers at the health center, at the sites you work at, or in Connecticut that might impact Spanish-language-preferred patients’ care experience?
   1. For example, economic barriers like insurance or payment, difficulty accessing care or treatment, fear or concern about stigma or discrimination?
6. How might health laws and regulations (at the health center or outside) impact patients who are most comfortable speaking in a language other than English?

**WRAP UP:**

1. Is there anything else you would like to share about your experience caring for/interacting with Spanish-language-preferred patients with chronic pain?

## Patient Interview Guide

Patient interviews assessed patients’ personal experience with chronic pain (microsystem), their interaction with their primary care provider and care team (macrosystem), and the factors at the health center (exosystem) and in the external environment (macrosystem) that they felt impacted their care experience.

**Open-Ended Questions:**

**1. Are you comfortable talking about your pain with your care team at CHC?**¿Se siente cómodo hablando de su dolor con su equipo médico en Community Health?
(su doctor, su enfermera, la asistente médico…)

Does your primary care provider speak Spanish? Do any other members of your care team?
¿Su proveedor medico habla español? Hay algunos otros miembros de su equipo medico quienes hablan español?

a. Describe what it is like to talk to your care team about the physical symptoms of your pain.
¿Cómo se siente cuando habla con sus médicos sobre los síntomas de su dolor?

b. Describe what it is like to talk to your care team about how pain has affected your life.
¿Cómo se siente cuando habla con sus médicos sobre cómo el dolor ha afectado su vida?

**2. How well does your care team at CHC understand what you have to say about your pain?**¿Usted cree que su equipo médico entiende lo que usted le dice sobre su dolor?

**3. How useful is the information your care team gives you about your pain and how to treat it?**Usted cree que la información que le da su equipo médico lo ayuda a entender su dolor y cómo aliviarlo?

(Probe: *What has helped you manage your pain? What has not helped?)*¿Qué le ha ayudado a aliviar su dolor? ¿Qué no le ha ayudado?

**4. What recommendations do you have to help CHC improve care for Spanish-speaking patients with chronic pain?**

Tiene algunas recomendaciones para que nosotros le demos al equipo para mejorar el cuidado de pacientes que hablan español y viven con dolor crónico.

1. These answer choices come from the Pew Research Center’s 2015 National Survey of Latinos (<https://www.pewresearch.org/hispanic/2017/12/20/methodology-hispanic-identity>) [↑](#footnote-ref-1)
2. Proficiency categories based on Interagency Language Roundtable Scale (ILR), developed by the U.S. Foreign Service Institute: S1 - Elementary Proficiency, S2 - Limited Working Proficiency, S3 - Professional Working Proficiency, S4 - Full Professional Proficiency, S5 - Native or Bilingual Proficiency (<https://www.icls.edu/foreign-language-programs/ilr-proficiency-levels/>) [↑](#footnote-ref-2)
