# Supplementary material for: A qualitative assessment of factors contributing to Spanish-speaking federally qualified health center patients’ chronic pain experiences
Source: PLoS One. 2023 May 18;18(5):e0285157. doi: 10.1371/journal.pone.0285157 (PMC10194871; doi:10.1371/journal.pone.0285157)
Supplement: S2 Appendix — (DOCX) [file pone.0285157.s002.docx]

**Appendix 2: Themes and example quotations from FQHC staff and patient interviews**

| **Category** | **Operationalized As** | **Themes and Subthemes** | **Example Quote** |
| --- | --- | --- | --- |
| **Microsystem:  Patient Level** | The interviewee's own beliefs, values, attitudes, knowledge, and behaviors | **Expressing Pain** | |
|  |  | Acknowledging Physical Pain | *"I think that there is a different affect to [Spanish-speaking Latinx patients'] pain expression, and I don’t know that that can be separated from language and culture. I do think that different cultures express pain in different ways, [and] there’s different degrees of being very expressive versus very stoic."* **[Staff #5]** *"Y ya tú te imaginas. Yo tengo ese dolor y me acuesta en la cama y no puedo hacer nada a veces...Esta bien mi hija yo lo dejo en las manos de ‘Dios.’"* **[Patient #12]** *"And so you can already imagine. I have this pain and I lay down on my bed and I cannot even do anything at times...It’s alright girlfriend I just leave it in the hands of ‘God.’"* **[Patient #12]** |
|  |  | Difficulty Locating or Describing Pain | *"I think descriptors of pain are a little bit easier in English, and I find it easier to get exactly the details that I need: how long, what does it feel like, where does it happen, when does it occur? … And then in Spanish, things [are] a little more amorphous… and sometimes at the end of the visit I’ll feel like I never really pinpointed exactly what they’re feeling. I know where it’s happening, kind of when it’s happening, but descriptors are a little different.*" **[Staff #6]** "Y*o lo que quiero es quitarme este dolor. Yo le cogi un poquito de miedo a la operación de la rodilla… no es nada más en la rodilla. La espalda, las caderas… Tengo dolor en todas las coyunturas."* **[Patient #9]** *"What I want is to rid myself of this pain. I was a bit scared by the knee operation...it's no longer the knee. My back, my hips...I have pain in all of my joints."* **[Patient #9]** |
|  |  | Vocabulary of Pain | *"I thought the biggest challenge was that [my patient] didn’t have enough words to describe what she was experiencing. The words she had were in Spanish, and some of these words you can’t translate, like, ‘yo tengo una pena’. If you translate what a ‘pena’ is, it’s ‘I have a sorry’."* **[Staff #2]**  *"Pain is such a nuanced thing and, you know, it feels different to me whether I have a Puerto Rican Spanish speaking person or a Guatemalan, or Mexican... how they describe pain [or] the experiences that caused the pain."* **[Staff #7]** |
|  |  | **Patients' Pain Experience** | |
|  |  | Attitude towards Treatment | *"Sometimes [my Latinx patients] are more focused on current symptoms right away and they’re not as open to exploring other factors impacting their pain experience such as psychological factors. And sometimes they may even be offended by the suggestion that it's good to have a conversation with a behavioral specialist."* **[Staff #9]** *"Es que ellos no hacen nada. Lo que me dan es ibuprofen..." [Later in interview, when asked how to improve care for Spanish-speaking patients with pain] "Bueno [recomiendo] que me receten la pastillas que es pal’ dolor."* **[Patient #10]** *"It's that they don't do anything. They give me ibuprofen..." [Later in interview, when asked how to improve care for Spanish-speaking patients with pain] "Well, [I recommend] that they prescribe me pills for the pain."* **[Patient #10]**  *"Para decirte la verdad es que yo he ido a sala de emergencia...Me recetan “painkillers’ - tres o cuatro pastillas y después tengo que ver mi doctor primario. Quiere que yo vaya hacer la terapia física, yo no quiero."* **[Patient #9]** *"To tell you the truth, I have gone to the emergency room... They prescribe me 'painkillers' - three or four pills and afterwards I have to see my primary care doctor. He wants me to go to physical therapy but I don't want to."* **[Patient #9]** |
|  |  | Home Remedies | *"In some cultures the management of illness and its symptoms can involve beliefs, foods, or natural products. I see that more frequently in my Spanish-speaking slash Latino population versus, for example, the white population."* **[Staff #9]**  *"Yo me unto - mi mai’ me lo manda de Puerto Rico - en la espalda que se llama manteca de ubre. Y eso es lo que me unto en la espalda pa’ dormir."* **[Patient #13]** *"I spread on myself - my mom sent it to me from Puerto Rico - on my back, it's called 'udder butter'. And that's what I spread on my back to sleep."* **[Patient #13]** |
|  |  | Pain and Behavioral Health | *"The pain increases the depression, the depression increases the pain. So they're definitely intertwined. For me it's more useful to think of them connected rather than which is first... we need to treat [both] together at the same time."* **[Staff #7]** *"Mira mi amor, ellos lo que hacen es que me mandan a [mental health treatment] y nada más. Y me dan pastillas…[* *Me gustaría] que se interesan un poco más por los pacientes."* **[Patient #7]** *"Look my love, what they do is they send me to [mental health treatment] and nothing more. And they give me pills... [I wish] that they were a little more interested in the patients."* **[Patient #7]** *"Al principio pues por lógica uno cree que uno va a coger un psicólogo porque está loco. ¿Verdad? A nadie le gusta ir a un psicólogo. Ahora después comprendí pues que me iba a servir para que me orientara un poco."* **[Patient #2]** *"At the beginning, logically, one thinks that one has to see a psychologist because they're crazy. Right? No one likes to go to a psychologist. But later I understood that it was going to help me orient myself a bit."* **[Patient #2]** |
| **Mesosystem: Care Team Level** | The interviewee's perception of interactions between patient and staff member regarding pain care, and patients' beliefs, values, attitudes, knowledge | **Empathy** | |
|  |  | Empathy | *"I think that [patients] just want to feel heard and taken care of, and sometimes giving a high score, or expressing your pain really dramatically, like, is like a manifestation of just wanting to be heard, kind of. And over time, I’ve definitely realized that just talking with people is a big part of chronic pain treatment. And hearing their – witnessing their pain and listening and validating it."* **[Staff #5]** *" Ellos no les interesa si tú te sientes bien, si tú te sientes mal… con una condición… con el diagnóstico que están dando…Cuando ellos me da el diagnóstico [of fibromyalgia] entonces es que, ‘oh, es como tu tienes una simple gripita – esto te va a pasar mañana' si estuviera normal, entiendes?"* **[Patient #2]** *"They aren't interested in whether you feel good or bad about the diagnosis that they're giving... When they gave me the diagnosis [of fibromyalgia], then it's like 'oh, it's like you have a simple cold - this is going to happen to you tomorrow' as though it was normal, right?"* **[Patient #2]  "***Me gustaría que me haga caso. To pay attention. Y le haga caso a lo que yo digo. I would like to have a doctor that makes me feel comfortable and understands where I am coming from. Don’t ignore [me] porque este dolor que estoy sufriendo de año y año."* **[Patient #12]** *"I would really like them to pay attention to me. To pay attention. And to pay attention to what I say. I would like to have a doctor that makes me feel comfortable and understands where I am coming from. Don't ignore [me], because I'm suffering this pain year after year."* **[Patient #12]** |
|  |  | **Mutual Understanding** | |
|  |  | Alignment between Patient and Provider | *"[Patients] have so many other needs that they have de-prioritized because of pain...diabetes follow-up, blood pressure follow-up. Chronic care that might be needed like mammograms, or cervical cancer, or colonoscopy. And all that kind of becomes de-prioritized because they are really [focused on] pain control."* **[Staff #4]** *"I would ask, ‘Are you sure, first of all, that you took that medicine? And for how long?’ I think she felt like she was definitely being questioned about, like, is she doing what I ask, and is her pain even real, when I’m saying ‘Are you sure it didn’t help?"*  **[Staff #6]** *"Seguramente tienes un dolor generalizado y tu cansancio que no sabe ni dónde viene, duermes poco... Pero, si, me ha costado… para que me creen me ha costado para que llegue por lo menos me creen."* **[Patient #2]** *"Surely you have a generalized pain, and your fatigue that you do not even know where it comes from, you sleep little... But yes it has cost me... in order for them to believe me it has cost me to arrive here, but at least they believe me."* **[Patient #2]** |
|  |  | Cross-Linguistic Communication | *"[Patients would] sometimes interrupt [the interpreter service] and be like 'that wasn't what I said' because they kind of knew English and Spanish.... When they felt like 'no interpreter, you got that wrong', I'm sure [it] really shook their confidence in their ability to communicate effectively with me."* **[Staff #1]** |
| **Exosystem:  Organization-Level** | The interviewee's experience of how patients with chronic pain are cared for at the organization | **Improving Access and Quality through Resources and Staffing** | |
|  |  | Interpretation Services | *"Aunque lo han mejorado mucho con el sistema que han puesto de ponerle traductor a uno…yo diría que sí que le tienen que poner un poquito de atención y empeño a eso."* **[Patient #2]** *"Even though they have improved a lot with the system that they put in place to translate for someone... I would say that they have to put a little attention and effort into that.*" **[Patient #2]** *"In terms of the language, there’s an impact of using translators…Usually when we're using the translator, the idioms of distress get lost."* **[Staff #9]**  *"[The patient] wanted someone to do an in-person visit, and specifically wanted a Spanish-speaking provider, because they didn’t want an interpreter listening to their story. I think there’s something that feels like ‘oh, this person is listening in to this’ that can be kind of a barrier."* **[Staff #5]** |
|  |  | Spanish-Speaking Personnel | *"I've had patients who are like 'get me somebody who speaks Spanish if you really want me to talk. I don't wanna talk to that machine [telephone translation service].'"* **[Staff #2]** *"I need a Spanish-speaking medical assistant, I need a Spanish-speaking nurse, and I don’t have that. And that’s tough, right? [The primary care provider is] just one piece of the puzzle."* **[Staff #6]**  "*Me siento cómoda… este… orgullosa porque mi doctora me atiende*. [When asked if their rating is related to the fact that the provider speaks Spanish] *"Sí. Claro que sí."* **[Patient #3]** *"I feel comfortable... I feel... proud because my doctor attends to me.* [When asked if their rating is related to the fact that the provider speaks Spanish] *"Yes, of course.”* **[Patient #3]** |
|  |  | Time Constraints | *"Ideally we could have more time with the translation service. [But] I think what people don’t always appreciate is that time you give to one patient is time you’re taking from another. There’s not more than X hours in a workday. So, if we give a subset of patients 40 minutes [instead of 20 minutes], we’ve taken a 20 minute appointment away from somebody else."*  **[Staff #4]** |
| **Macrosystem: Systems and Policy Level** | The interviewee's knowledge of and perspectives on the influence of health policy and social determinants of health on how patients are cared for | **Culture** | |
|  |  | Approach to Pain Treatment | *"I love the fact that I am able to connect with [patients] enough to explain that there are other [non-opioid treatments] that can mitigate their pain based on the other things in their life and how they’re feeling - whether it’s their anxiety, their depression, their experience with other supports in their house and other relationships that they have."* **[Staff #1]** |
|  |  | Diversity among Spanish-Speaking Patients | *"It’s not just being able to speak Spanish, it's also being able to understand different cultures , because it's not a monolithic population...Spain is not the same as Mexico is not the same as Guatemala, or Puerto Rico, so it's just understanding differences, even if you have a common language."* **[Staff #8]** |
|  |  | Need for Linguistic and Cultural Concordance and Competence | *"I have a lot of people that specifically request a Spanish-speaking provider. They just like the feeling of being able to speak to someone in their native language."* **[Staff #6]**  *"I feel very comfortable [treating patients with pain]... as I share cultural background with them...I can understand their idioms, for example, and that helps… in the rapport building... Our experiences and culture impact everything in our lives - how we understand what is happening to us and why it is happening and how we explain it and how we describe the symptoms."* **[Staff #9]**  *"I think it's [within] our ability as a healthcare field to be able to understand how different cultures define or express pain... I just think that sometimes our understanding of chronic pain, and how patients are able to express it - there might be some misalignment in how we're able to perceive and/or diagnose chronic pain based on current definitions."* **[Staff #8]** |
|  |  | **Social Determinants of Health** | |
|  |  | Financial, Occupational, and Social Stressors | *"But the patients we see at [site] who are from other countries and undocumented often work 3 or 4 jobs… and it’s often physical jobs, kind of similar to the farmworker population. And so chronic pain is also a really common issue"* **[Staff #5]** *"Claro. Yo era responsable [para mi dolor]. Trabajaba en construcción...por mas de treinta [años]"* **[Patient #9]** *"Of course. I was responsible [for my pain]. I worked in construction... for more than thirty [years]."* **[Patient #9]** |
|  |  | Health Disparities | *"I’d also say [patients’] access to food, and particularly good nutrition is challenging. So not only do they have jobs that have more wear and tear on their bodies, but they tend to really struggle with obesity and being overweight, a lot of it because of their diet, nutrition, and sometimes even inability to exercise or to feel safe in the environment that they’re living with in order to exercise... There’s just this whole constellation of things."* **[Staff #1]** |
|  |  | Information and Resources | “*There's yoga, I have a massage therapist, I go to a chiropractor… But I have access to these things. Most of our patients don't. Most patients [tell me they] don't have 25 bucks to go to a damn yoga class, right?*" **[Staff #2]**  *"You should ask Spanish language-preferred patients what can be done to improve. I know we have a third party that does patient satisfaction surveys, but [we don't do] enough, I think in terms of...directly asking patients. And then also asking our own staff about what are they hearing from patients...staff at all levels who are part of the healthcare team or [FQHC non-clinical] staff. "* **[Staff #8]** |
